# Immunopathology of Acute Kidney Injury in Severe Malaria

**DOI:** 10.3389/fimmu.2021.651739

**Published:** 2021-04-23

**Authors:** Orestis Katsoulis, Athina Georgiadou, Aubrey J. Cunnington

**Affiliations:** ^1^ Section of Paediatric Infectious Disease, Department of Infectious Disease, Imperial College London, London, United Kingdom; ^2^ Centre for Paediatrics and Child Health, Imperial College London, London, United Kingdom

**Keywords:** malaria, inflammation, acute kidney injury (AKI), immune response, hemolysis, *P. falciparum*, kidney injury

## Abstract

Acute kidney injury (AKI) is a common feature of severe malaria, and an independent risk factor for death. Previous research has suggested that an overactivation of the host inflammatory response is at least partly involved in mediating the kidney damage observed in *P. falciparum* patients with AKI, however the exact pathophysiology of AKI in severe malaria remains unknown. The purpose of this mini-review is to describe how different aspects of malaria pathology, including parasite sequestration, microvascular obstruction and extensive intravascular hemolysis, may interact with each other and contribute to the development of AKI in severe malaria, by amplifying the damaging effects of the host inflammatory response. Here, we highlight the importance of considering how the systemic effects and multi-organ involvement of malaria are intertwined with the localized effects on the kidney.

## Introduction

Malaria is one of the most common infectious diseases in the world and one of the most significant challenges faced by public health systems in many developing countries. An estimated 229 million cases of malaria and 409,000 malaria deaths were reported worldwide in 2019, with the overwhelming majority of cases being reported in Africa, and children accounting for approximately 67% of worldwide deaths (1). The COVID-19 pandemic has posed a worrying threat to already fragile malaria control programs, by further burdening the healthcare systems of malaria-endemic countries (2).

Malaria is transmitted through the bite of a *Plasmodium* spp.-infected female *Anopheles* mosquito, which inadvertently provides the parasite with access into the human host (3). The clinical spectrum of infection with these parasites can range from asymptomatic, to uncomplicated malaria, characterized by non-specific paroxysmal symptoms such as fever, headache and muscle ache, to severe malaria (SM), characterized by life-threatening symptoms, including severe anemia, coma, acute kidney injury and metabolic acidosis (4).

Acute kidney injury (AKI) is defined as an abrupt and rapid deterioration in kidney function, and it represents one of the most serious complications of severe *P. falciparum* infection in humans, having been repeatedly associated with higher patient mortality. The KDIGO (Kidney Disease: Improving Global Outcomes) classification currently defines AKI as either: 1) an increase in serum creatinine by at least 0.3 mg/ld. within 48 hours, or 2) an increase in serum creatinine to at least 1.5 times the baseline level within the previous 7 days, or 3) a decrease in urine output to less than 0.5 mL/kg/h for 6 hours (5). AKI is a multifactorial condition with many potential risk factors, while the causes of AKI are usually categorized as prerenal (including hypovolemia and obstruction of blood flow), postrenal (obstruction of urinary flow) and renal (including nephrotoxins, infection, and inflammation) (6). Prior to the use of the KDIGO consensus guidelines, the prevalence of AKI in malaria had been significantly underrepresented in both adults and children (7, 8). Recent studies using the KDIGO classification, have reported that the prevalence of AKI ranges from 20% to 40% among adults and children with SM, while some studies have reported an AKI incidence of as high as 59% for children (9–14). Despite the similar frequency of AKI between child and adult SM patients, the overall burden of malaria-associated AKI cases is likely to be greater in children, since the vast majority of SM patients in endemic countries are children (15). Importantly, kidney injury has been found to be an independent predictor of mortality in children with SM, though it is not yet known if and how AKI directly contributes to death (16). At the same time, a study examining long-term clinical outcomes of malaria-related AKI patients, showed that 5% of patients developed chronic kidney disease, further highlighting the significance of AKI as a complication of SM (17).

Typical histopathology features in malaria-related AKI include acute tubular necrosis (ATN) and, less commonly, interstitial nephritis and glomerulonephritis (18, 19). Examination of kidney tissues from autopsies of Southeast Asian adult SM patients revealed the presence of sequestered pRBCs within glomerular and tubulo-interstitial vessels, as well as the accumulation of host monocytes within glomerular and peritubular capillaries (20). Increased glomerular cell proliferation and decreased expression of zonula occludens-1 protein (ZO-1) has also been observed in kidney tissue from malaria-related AKI patients compared to non-AKI SM patients (21).

The pathogenic mechanisms of AKI in malaria are not yet fully defined, although there are multiple pathological processes which may converge on the kidney, including parasite sequestration, endothelial dysfunction, oxidative stress and immune-mediated damage. One of the hallmarks of malaria infection is intravascular hemolysis, primarily of *Plasmodium*-infected red blood cells (pRBCs), which leads to the release of cell-free heme, as well as both host and parasite-derived molecules that potently trigger inflammatory responses (4). Some degree of intravascular hemolysis occurs with all parasite species, but the most extensive hemolysis occurs in *P. falciparum* infection, as a consequence of the higher parasite densities typically occurring in the blood with this parasite species (22). The particular virulence of *P. falciparum* is also attributed to expression of parasite proteins on the surface of pRBCs, which allows their adherence to the endothelium of blood vessels, through binding to endothelial receptors (e.g. ICAM-1, EPCR) (23). Sequestered pRBCs evade splenic clearance, contributing to high parasite load, obstruct small blood vessels, leading to tissue hypoxia, and activate vascular endothelial cells (4). Endothelial activation is likely a central pathological event, resulting in impairment of its barrier function, dysregulation of blood flow and coagulation cascades, and secretion of proinflammatory cytokines, further amplifying the host inflammatory response (3).

**Figure 1:**
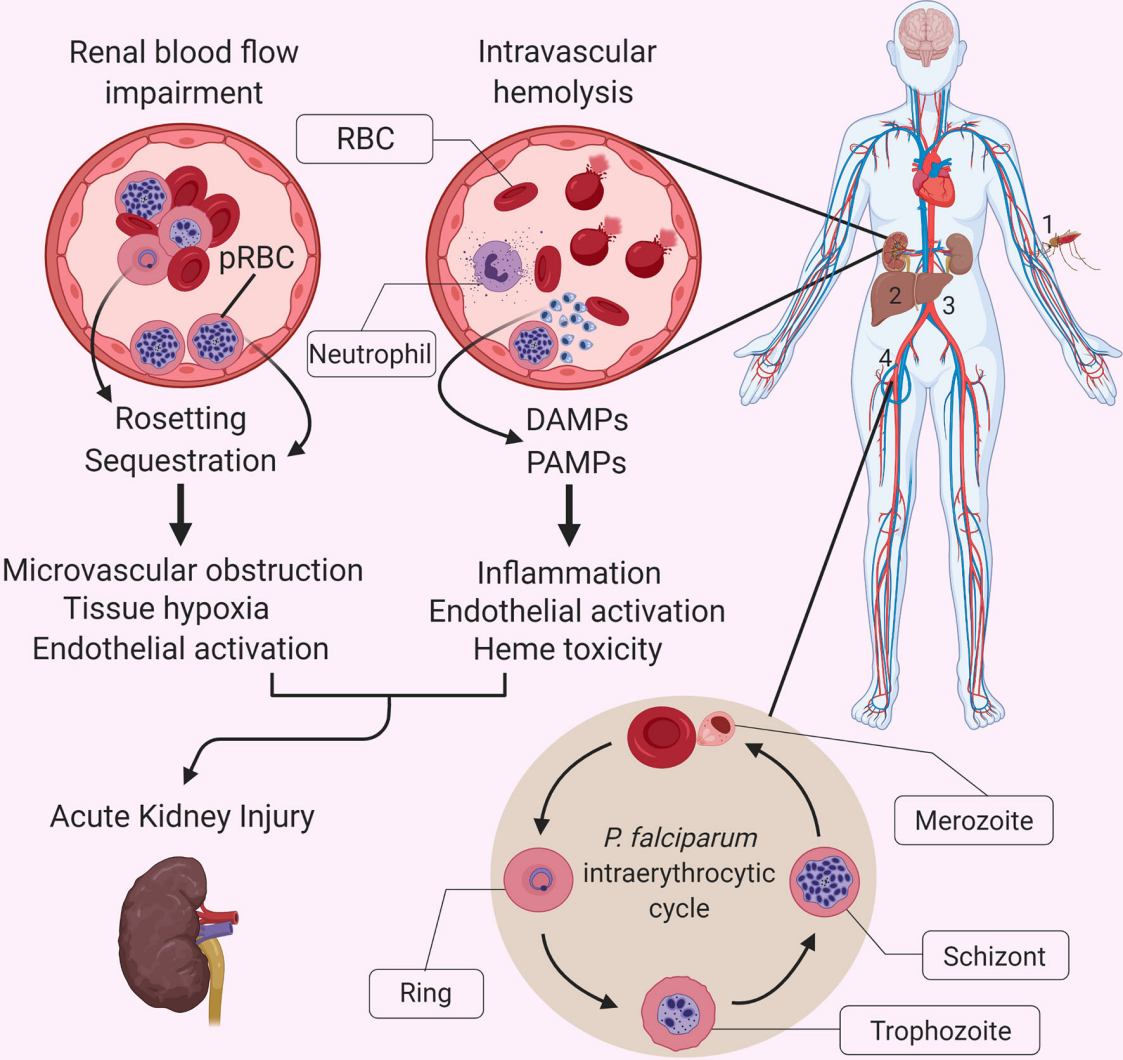
Interactions between *P. falciparum* malaria-specific pathology and the systemic inflammatory response in malaria-related acute kidney injury. The causative agents of malaria are protozoan parasites belonging to the Plasmodium genus, which includes multiple species, five of which regularly infect humans: *P. falciparum, P. vivax, P. ovale, P. malariae* and *P. knowlesi* (4). A bite by a *Plasmodium* spp.-infected mosquito (1) leads to the injection of motile sporozoites within the host, which travel through the lymphatics and blood until they reach the liver and invade hepatocytes (2). Once inside the hepatocyte, each sporozoite replicates and gives rise to thousands of merozoites, which are released into the bloodstream (3) when the infected hepatocyte bursts. Free merozoites then proceed to infect red blood cells (RBCs) within the bloodstream (4) and enter their asexual reproductive cycle, also referred to as the intraerythrocytic cycle. This developmental stage is characterized by a replication cycle that typically lasts approximately 48 hours and which culminates in the simultaneous rupturing of the parasitized RBCs (pRBCs), and the release of a massive number of merozoites into the bloodstream, which go on to infect further RBCs. Malaria is associated with a vigorous inflammatory response, which shares some features with other infectious diseases, but it is accompanied by unique aspects of pathophysiology that exacerbate the impact of systemic inflammation on individual organs. These unique aspects include the cytoadherence of parasite-infected red blood cells (pRBCs) to the microvascular endothelium, and the extensive release of cell free hemoglobin and heme during hemolysis. Although the exact pathophysiology of AKI in severe malaria remains unknown, we propose that the sequestration of parasitized red blood cells, rosetting and accumulation of parasite products in the kidney, resulting in endothelial activation and microvascular obstruction, promote the already damaging effects of an exuberant inflammatory response and heme-mediated oxidative damage. DAMP, Damage-associated molecular pattern; PAMP, Pathogen-associated molecular pattern.

The synergistic occurrence of the unique features of SM pathology, including parasite sequestration, microvascular dysfunction, endothelial activation, extensive intravascular hemolysis and hemodynamic instabilities, culminating in the exacerbation of a vigorous systemic inflammatory response on the kidneys, could represent the predominant mechanism through which SM leads to the development of AKI (**Figure 1**). Understanding the role of the inflammatory response and the extent to which it contributes to malaria-associated AKI, could lead to the development of novel immunomodulatory therapies that significantly decrease the number of deaths caused by both malaria-related and other causes of AKI.

## Immune and Inflammatory Responses in Malaria

The host immune response has been implicated as a detrimental factor in the pathogenesis of SM, wherein an exaggerated proinflammatory response against the parasite is thought to contribute to the pathology of the disease (3). The intravascular hemolysis of both pRBCs and non-infected RBCs leads to the release of parasite and host-derived molecules that act as pathogen-associated molecular patterns (PAMPs) and damage-associated molecular patterns (DAMPs), respectively (24). These PAMPs and DAMPs interact with pattern recognition receptors (PRRs), such as toll-like receptors (TLRs) and NOD-like receptors (NLRs), which activate multiple downstream transcriptional programs related to the host immune and inflammatory response. Glycosylphosphatidylinositol (GPI) anchors are one of the main *Plasmodium* PAMPs that act through the activation of TLRs, inducing the production and secretion of proinflammatory cytokines such as tumor necrosis factor (TNF) and the expression of cell-adhesion molecules (e.g. VCAM-1) (25). Another major PAMP is hemozoin, a molecule produced by the *Plasmodium* parasite within RBCs during the detoxification of heme. Hemozoin crystals elicit the production of TNF and IL-1β by monocytes and macrophages, while hemozoin complexed with *Plasmodium* nucleic acids can further amplify the inflammatory response by interacting with cytosolic DNA sensors, leading to the activation of inflammasomes (e.g. NLRP3, AIM2) (26). Host-derived DAMPs are also released during intravascular hemolysis, with cell-free hemoglobin and heme considered to be the predominant ones. Although the activation of these PRRs is vital for the mounting of a protective immune response against the parasite, the constant and excessive activation that occurs during malaria due to continuous release of PAMPs and DAMPs leads to a state of systemic hyperinflammation, which may contribute to disease pathology.

SM patients have been found to produce greater levels of proinflammatory cytokines (e.g. IL-1β, IL-6 and TNF), than patients with uncomplicated malaria, while evidence has accumulated over the years implicating the upregulation of the inflammatory response as a contributory factor to SM pathogenesis, and malaria-associated AKI in particular (27, 28). For instance, macrophage infiltrates have been observed in the glomeruli of *P. berghei* ANKA-infected mice, which were accompanied by a strong upregulation in the expression of proinflammatory cytokines in kidney tissue, including TNF and IL-6 (29). Both TNF and IL-6 signaling can potently propagate the inflammatory response and induce activation of the endothelium, while TNF has also been shown to cause changes to the glomerular endothelial permeability (30, 31). Endothelial activation induces an upregulated expression of cell adhesion molecules on the surface of endothelial cells (e.g. VCAM-1, ICAM-1, E-selectin), which could in turn lead to increased sequestration of pRBCs and infiltration of leukocytes into the kidneys, thus contributing to AKI in malaria. Multiple studies have concluded that markers of endothelial activation and microvascular obstruction are associated with malaria-related AKI in both children and adults with SM (32–34).

Activation of the complement system has also been suggested to contribute to kidney damage in cases of intravascular hemolysis (35). The liberation of hemoglobin and its breakdown products potently activates the alternative complement pathway, which then induces opsonization of both infected and uninfected RBCs, further exacerbating the intravascular hemolysis that characterizes malaria (36, 37). Activation of the alternative complement pathway has been repeatedly shown to contribute to tubular injury and kidney function deterioration in mouse models of ischemia/reperfusion injury-related AKI, while selective inhibition of the pathway significantly reduced degree of kidney injury (38, 39). Importantly, neutrophils activated by proinflammatory cytokines such as TNF, are known to be potent inducers of the alternative complement pathway (40). At the same time, activated complement goes on to further propagate the activation of neutrophils, thus creating a positive feedback loop that amplifies both the neutrophil proinflammatory response and the activation of the complement system, which can be detrimental for the kidneys (40, 41).

## Hypovolemia and Obstruction of Kidney Blood Flow

The kidneys are especially susceptible to the damaging effects of ischemic events and hemodynamic instabilities, and obstruction of blood flow into the kidneys represents one of the primary causes of AKI (5). Significant hypovolemia is a common phenomenon in SM, often leading to kidney hypoperfusion, decreased GFR, secretion of vasoactive mediators and the activation of inflammatory processes, all of which could contribute to the development of kidney injury (28, 42). Recent evidence implicating hypovolemia-related inflammation in playing a role in the kidney damage associated with SM came from a study investigating the activation of the Angiotensin II (Ang II)/AT1 receptor pathway in the *P. berghei* ANKA malaria mouse model (28). Activation of this pathway has been found to play a central role in the occurrence of kidney injuries in multiple AKI mouse models, through its ability to increase the expression of proinflammatory cytokines and induce immune cell infiltration into tissues (28, 43). Importantly, inhibition of the Ang II/AT1 pathway in the *P. berghei* ANKA mouse model led to a significant downregulation of the inflammatory response and a substantial mitigation of kidney damage (28).

In addition to hypovolemia, kidney hypoperfusion in SM might result from obstruction of blood flow within the kidneys. During SM, clumps of both infected and uninfected RBCs, also referred to as ‘rosettes’, accumulate in the lumen of small blood vessels and obstruct blood flow, leading to microvascular dysfunction and tissue hypoxia, while also contributing to the sequestration of greater parasite biomass within tissues (3). High parasitemia and extensive sequestration of pRBCs in tissues and vital organs has been correlated with poorer outcomes in SM patients (44). Cerebral malaria (CM) has been associated with a high parasite burden in the brain and retinal tissue of patients (45). Similarly, total body parasite burden and immune activation were found to be associated with the incidence of AKI in Bangladeshi patients with SM, while both factors increased with AKI severity (46). Extensive sequestration of parasites within the kidney could elicit a stronger local inflammatory response, which may in turn exacerbate the degree of kidney injury.

## Extensive Intravascular Hemolysis and Heme-Mediated Toxicity

Both cell-free hemoglobin and cell-free heme released during intravascular hemolysis can be particularly harmful, mainly due to oxidative stress and by acting as DAMPs that stimulate the immune system and lead to an upregulation of the inflammatory response (47). The body possesses natural scavenging systems in order to clear cell-free hemoglobin and cell-free heme, but these systems can be quickly overwhelmed by extensive hemolysis such as that observed during SM (35). Furthermore, the kidneys are especially vulnerable to the damaging effects of cell-free heme and hemoglobin, since they are the primary route for clearance of these molecules when the scavenging systems become saturated (35).

Both infectious and non-infectious causes of extensive intravascular hemolysis have been associated with the development of AKI, while increased levels of plasma and urine hemoglobin have also been strongly associated with AKI in SM patients (35, 48). Higher levels of cell-free hemoglobin and markers of lipid peroxidation have been observed in SM patients with AKI compared to those without AKI (48). A recent clinical trial showed that SM patients receiving acetaminophen, a drug which can inhibit hemoprotein-mediated lipid peroxidation, had a lower risk of developing AKI than patients in the control group. The effect of acetaminophen was most prominent in patients with greatest intravascular hemolysis, further strengthening the association of cell-free hemoglobin and oxidative stress with AKI (49).

Past studies have investigated the effect of sterile intravascular hemolysis on the kidneys by treating mice with phenylhydrazine (PHZ), a chemical compound that induces massive intravascular hemolysis (47). One of these studies found that kidney endothelial cells from mice treated with PHZ had increased expression of cell adhesion molecules (e.g. E-selectin and ICAM-1), while they also detected markers and ultrastructural signs of tubular damage (47). In the context of SM, this upregulation of adhesion molecules by the endothelium could significantly worsen the extent of parasite sequestration within tissues, as well as increase the infiltration of inflammatory immune cells into the kidneys, as previously discussed (47). Interestingly, the same study showed that the injection of heme alone did not recapitulate this effect of tubular injury and vascular inflammation, supporting the notion that it is a combination of factors and mechanisms acting in concert that lead to the development of both malaria-related and non-related AKI, rather than one individual mechanism (47).

Kidney endothelial cells exposed to cell-free heme *in vivo* have been found to upregulate their secretion of proinflammatory chemokines, such as monocyte-chemoattractant protein 1 (MCP-1), through the activation of the NFκB transcription factor by heme (50, 51). Intriguingly, a recent study showed that a mouse model of malaria with a renal proximal tubule-specific knockout of heme oxygenase-1 (HO-1), a heme detoxifying enzyme, was more susceptible to AKI, further suggesting the involvement of heme-mediated damage in the propagation of AKI in SM (52). Additionally, the same effect was found when the researchers knocked out ferritin, which is responsible for sequestering the labile iron released after the detoxification of heme by HO-1, strengthening the association of oxidative stress with kidney injury (52). Multiple pieces of evidence have indicated the involvement of heme oxygenase-1 (HO-1) and the by-product of heme degradation, carbon monoxide (CO), to be intricately involved in the establishment of disease tolerance to SM complications, through their antioxidant and anti-inflammatory properties (52). Carbon monoxide therapy, administered as carbon monoxide-releasing molecules, has been reported to confer full protection against experimental cerebral malaria and acute lung injury in a mouse model when used in conjunction with an antimalarial drug (53). Since the beneficial impact of CO is thought to be thanks to its ability to prevent the further release of heme from hemoglobin and to induce the expression of HO-1, it is possible that there would be a therapeutic potential against malaria-associated kidney damage.

Finally, cell-free heme is known to trigger neutrophil activation, inducing the release of neutrophil extracellular traps (NETs). In a study using *P. chabaudi*-infected mice, heme-induced NET generation was found to be necessary for the development of liver and lung pathology and also increased the extent of parasite sequestration in the organs of the mice (54). If the same phenomenon of heme-induced NET release were to occur in the kidneys, it would provide another mechanism through which intravascular hemolysis might lead to immune-mediated kidney damage. NETs have also been implicated as a contributing factor in non-malaria related causes of AKI, including systemic lupus erythematosus (SLE) and ANCA-associated vasculitis (55). Furthermore, the release of histone proteins from necrotic kidney tubular cells during ischemic AKI is known to induce the formation of NETs, and in a study utilizing an ischemia-reperfusion injury mouse model of AKI, pre-treatment of the mice with an inhibitor of NET formation led to a mitigation of kidney injury (56). NETs might also be involved in further obstructing microvascular blood flow in cases of ischemic AKI, by creating thrombus-like structures made up of trapped cells and cellular debris (57). In addition, the formation of NETs may represent a proinflammatory stimulus by itself, since the histone proteins and DNA that are released during the process can act as DAMPs that further activate the immune response (57). Immunofluorescence studies to detect neutrophil infiltration and the presence of NETs in kidney tissue from mouse models of malaria could prove useful in the future, to determine whether NETs are also involved in mediating kidney pathology during malaria-related AKI.

## Discussion

Multiple studies have identified malaria as one of the most frequent etiologies of pediatric AKI cases in developing countries (58). Importantly, both adult and pediatric studies have shown that when AKI occurs in the context of malaria, there is an increase in patient mortality (59, 60). One of the key questions that remains to be answered is whether AKI plays a causal role in SM deaths, or if it is a consequence of pathological processes which lead to death through their effects on other organs.

**Figure 2:**
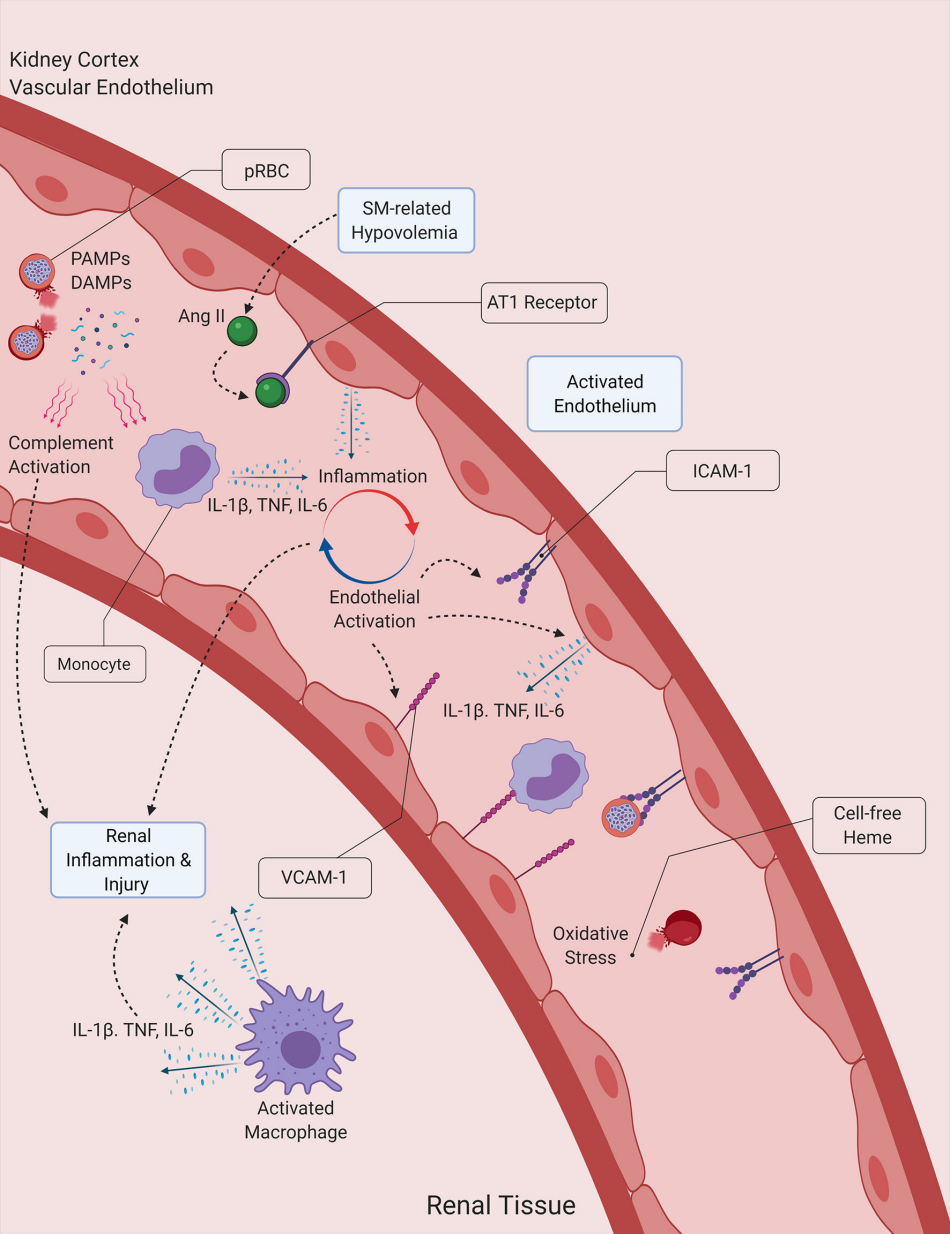
Proposed interactions between inflammation, hemolysis and hypovolemia in malaria-induced kidney injury. Malaria PAMPs and DAMPs released during the lysis of *Plasmodium*-infected RBCs (pRBCs) stimulate the immune and inflammatory responses, leading to the secretion of proinflammatory cytokines, which subsequently induce the activation of the endothelium and the propagation of the inflammatory process. Activated endothelial cells contribute to the secretion of proinflammatory cytokines, and express surface receptors that facilitate the infiltration of leukocytes into kidney tissue. Cell-free heme acts a source of oxidative stress for the vascular endothelium and the tubular epithelial cells of the kidney, while also inducing the formation of NETs. The activation of the angiotensin (Ang) II/AT1 receptor pathway due to malaria-induced hypovolemia also contributes to the amplification of the host inflammatory response, by further inducing the secretion of proinflammatory cytokines by endothelial cells. DAMP, Damage-associated molecular pattern ICAM-1, Intercellular adhesion molecule 1, IL-1β, Interleukin-1β; IL-6, Interleukin-6; NET, Neutrophil extracellular trap; PAMP, Pathogen-associated molecular pattern; SM, Severe malaria, TNF, Tumor necrosis factor; VCAM-1, Vascular cell adhesion molecule 1.

Here, we have described the factors and pathways plausibly involved in the pathophysiology of AKI in SM. It is hypothesized that a vigorous host inflammatory response which is amplified by features of malaria pathology appears to be a central aspect of kidney injury in SM. Hypovolemia and obstruction of kidney blood flow lead to tissue hypoxia and endothelial activation, which could promote the secretion of proinflammatory cytokines and leukocyte infiltration into the kidneys. At the same time, the cell-free heme that is liberated due to intravascular hemolysis may also contribute to kidney damage, not just by acting as a source of oxidative stress, but also by triggering the activation of the complement system, the formation of NETs and by further amplifying the proinflammatory response (**Figure 2**).

In light of the pivotal role that the inflammatory response is hypothesized to play in mediating kidney injury, it is imperative that we obtain a greater understanding of the immunopathology of AKI in malaria, which will hopefully lead to the development of more effective treatments and a decrease in the mortality of the disease.

## Author Contributions

AC and AG conceived the idea for this review. OK wrote the original draft and designed the figures of the manuscript. AC and AG provided critical feedback and reviewed the final version of the manuscript. All authors contributed to the article and approved the submitted version.

## Funding

Infrastructure support for this work was provided by the National Institute for Health Research (NIHR) Imperial Biomedical Research Centre (BRC). AG is supported by an Imperial College Research Fellowship.

## Conflict of Interest

The authors declare that the research was conducted in the absence of any commercial or financial relationships that could be construed as a potential conflict of interest.
